# Anti-ROR1 CAR-T cells: Architecture and performance

**DOI:** 10.3389/fmed.2023.1121020

**Published:** 2023-02-17

**Authors:** Daniel Andrés Osorio-Rodríguez, Bernardo Armando Camacho, César Ramírez-Segura

**Affiliations:** ^1^Laboratorio de Investigación en Ingeniería Celular y Molecular, Instituto Distrital de Ciencia, Biotecnología e Innovación en Salud (IDCBIS), Bogotá, Colombia; ^2^Instituto Distrital de Ciencia, Biotecnología e Innovación en Salud (IDCBIS), Bogotá, Colombia

**Keywords:** chimeric antigen receptor (CAR), receptor tyrosine kinase-like orphan receptor 1 (ROR1), solid tumors, hematologic tumors, cancer therapy, tumor marker

## Abstract

The receptor tyrosine kinase-like orphan receptor 1 (ROR1) is a membrane receptor that plays a key role in development. It is highly expressed during the embryonic stage and relatively low in some normal adult tissues. Malignancies such as leukemia, lymphoma, and some solid tumors overexpress ROR1, making it a promising target for cancer treatment. Moreover, immunotherapy with autologous T-cells engineered to express a ROR1-specific chimeric antigen receptor (ROR1 CAR-T cells) has emerged as a personalized therapeutic option for patients with tumor recurrence after conventional treatments. However, tumor cell heterogeneity and tumor microenvironment (TME) hinder successful clinical outcomes. This review briefly describes the biological functions of ROR1 and its relevance as a tumor therapeutic target, as well as the architecture, activity, evaluation, and safety of some ROR1 CAR-T cells used in basic research and clinical trials. Finally, the feasibility of applying the ROR1 CAR-T cell strategy in combination with therapies targeting other tumor antigens or with inhibitors that prevent tumor antigenic escape is also discussed.

**Clinical trial registration:**

https://clinicaltrials.gov/, identifier NCT02706392

## Introduction

According to the World Health Organization (WHO), cancer is the leading cause of death worldwide. Breast, lung, colon and rectum, prostate, skin, and stomach cancers were the most frequent solid neoplasms in 2020, with lung cancer showing the highest mortality (1). In turn, leukemia and non-Hodgkin’s lymphoma presented the highest mortality in the group of hematological tumors (2). Given the prevalence and mortality of cancer, it is pertinent to invest in new therapeutic alternatives for treating patients affected, especially those who experience relapses. Therapies for cancer management currently available include surgery aimed at removing as much of the tumor as possible, followed by chemotherapy and radiotherapy. In addition, some malignancies can be treated with endocrine therapy or small molecule inhibitors (3, 4). Mutations acquired by cancer cells change the normal gene expression profile (5, 6). Thereby, many malignant cells gain a stem cell profile and thus proliferate without restriction (7). In some cases, these changes lead to overexpression of membrane molecules, such as the receptor tyrosine kinase-like orphan receptor 1 (ROR1) and the epidermal growth factor receptor (EGFR), which are found in breast cancer, lung adenocarcinoma, and glioblastoma (8–12) and are considered promising therapeutic targets.

In both hematological malignancies and solid tumors, there is a complex scenario know as tumor microenvironment (TME) (13–16) that comprises other cell types (fibroblasts, immune cells and blood vessel cells), soluble factors, and components of the extracellular matrix (ECM). The interaction between all those components stimulates abnormal cell proliferation and promote the emergence of resistance mechanisms in tumor cells (16–18). In solid tumors, T-cell infiltration is fundamental for immunotherapeutic success. In this scenario, TME has attracted attention for creating a physical barrier that leads to insufficient accumulation of immune cells within the tumor. TME further inhibits tumor-infiltrating lymphocytes (TILs) due to the presence of immunosuppressive checkpoint molecules that bind the programmed death receptor 1 (PD-1) and cytotoxic T-lymphocyte-associated antigen 4 (CTLA-4), which in turn, suppress the immune response (16, 19). Inhibitory factors in the TME are therapeutical targets to reduce the immunosuppressive microenvironment (20). For example, monoclonal antibodies (mAbs) can bind to PD-1 (21, 22) or CTLA-4 (23) expressed on T-cells to block the respective downstream signaling pathways and restore the antitumor immune response. Stuber et al. (24) showed that the inhibition of TGF-β improved the effector activity of antitumor T-cells in a breast cancer model (24). Similarly, ECM plays a crucial role in tumor progression promoting malignant transformation [reviewed in (25, 26)], and ECM components are targeted to enhance tumor infiltration by immune cells. Examples are T-cells genetically engineered to secrete a heparanase capable of cleaving heparan sulfate (27) as well as target fibronectin to enhance T-cell infiltration (28). In addition, most therapies are administered intravenously and are effective against hematological malignancies but without similar efficacy in solid tumors; therefore, direct administration of antitumor T-cells has shown promising results in treating glioblastoma and ovarian cancer (29–31).

Furthermore, the tumor cell heterogeneity that induces resistance to conventional treatments and the occurrence of aggressive relapses become significant challenges to achieving a successful outcome (7). Other therapeutic options for cancer management are emerging, such as the immunotherapy with mAbs (32–37), bi-specific T-cell engagers (BiTEs), a kind of mAbs, which are fusion proteins containing two antibody binding sites, one for tumor antigen on cancer cells, and other for simultaneous binding to CD3 on T-cells (38); Blinatumomab (39) and Anti-ROR1 BiTE (40) belong to this category. Therapies with oncolytic viruses (41), such as Teserpaturev/G47Δ, have also been implemented. This product contains a recombinant herpes simplex virus type 1; it has been approved for the treatment of malignant glioma in Japan, and it is currently under clinical development for the management of prostate cancer (phase II), malignant pleural mesothelioma (phase I), and recurrent olfactory neuroblastoma (phase I) (42). Anticancer approaches based on microRNAs (43–46), TILs (47–49), and T-cells engineered to express a chimeric antigen receptor (CAR-T cells) against tumor antigens are other current alternatives for the treatment of cancer (50–52).

Chimeric antigen receptor therapy involves the *ex vivo* genetic manipulation of autologous T-cells to enable them to recognize a specific antigen present on the surface of malignant cells. The *CAR* gene, which encodes a single-chain variable fragment (scFv) specific for a tumor antigen and some costimulatory domains, is inserted into T-cells by laboratory techniques to be expressed on the T-cell membrane. Subsequently, the interaction between the CAR on T-cells and the tumor antigen on cancer cells triggers an immune response resulting in neoplastic cell death (53). The United States Food and Drug Administration (FDA) has approved the use of CAR-T cells targeting the cluster of differentiation 19 (CD19) (54–56) and the B-cell maturation antigen (BCMA) (57, 58) for the treatment of hematological malignancies. CD19 CAR-T cells and BCMA CAR-T cells were safe and effective in patients with refractory B-cell acute lymphoblastic leukemia (B-cell ALL) and multiple myeloma (MM), respectively. Although CAR-T cell therapy has shown good outcomes in patients with leukemia, lymphoma, and MM, it remains a challenge in the case of solid tumors, due to the immunosuppressive TME and the poor persistence of CAR-T cells *in vivo* (59–61).

## Discovery and biological function of ROR1

Receptors with tyrosine kinase activity and their ligands regulate several cellular processes, such as development and survival. However, they may also participate in the emerging of diseases, such as cancer [reviewed in (62)]. This receptor group includes the tyrosine kinase-like orphan receptors (RORs) family. The two members of this family, ROR1 and ROR2, are generally involved in signaling pathways of cell proliferation, differentiation, and migration, as well as angiogenesis, and play essential roles in the development of various tissues and organs (63–65), such as the skeletal and neural ones (66). Despite RORs belong to the tyrosine kinase family, they do not share a conserved amino acid sequence with other tyrosine kinases (63).

**FIGURE 1:**
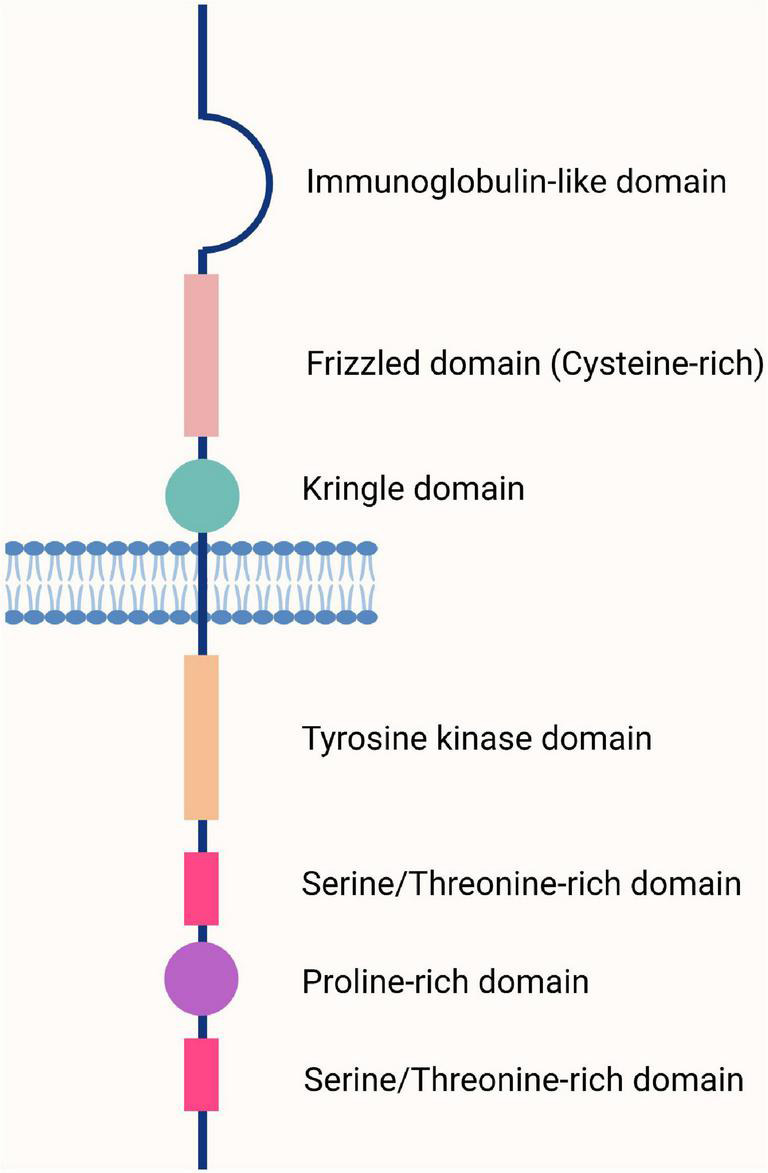
The basic structure of the human and mouse receptor tyrosine kinase-like orphan receptor 1 (ROR1) receptor. The ROR1 receptor comprises an extracellular region containing three domains (Ig-like, Frizzled, and Kringle), a transmembrane region, and a cytoplasmic region containing four domains (tyrosine, serine/threonine, proline, and serine/threonine). Adapted from Aghebati-Maleki et al. (70).

Receptor tyrosine kinase-like orphan receptor 1 and ROR2 were discovered during the search for tyrosine kinase coding genes. They share 58% of the amino acid sequence of tyrosine kinases (67). Interestingly, the sequence of ROR1 is highly conserved between humans and mice (97% similarity) (Supplementary Figure 1A); that of ROR2 reaches 92% (68). There are three ROR1 isoforms produced by alternative splicing (69) (Supplementary Figure 1B). The ROR1 isoform 1 (UniProt: Q01973) has an extracellular region with three domains: An immunoglobulin-like domain (P42-F147), a cysteine-rich domain (E165-I299, also called Frizzled domain); and a Kringle domain (K312-C391). The extracellular region is followed by a transmembrane segment that spans from I407 to V427, and the cytoplasmic region (C428-L937) that contains a tyrosine kinase domain (V473-L746) and a proline-rich domain between two serine/threonine-rich domains (69, 70) (Figure 1). The ROR1 isoform 2 (Q01973-2) comprises only the intracellular region (69, 71), whereas the ROR1 isoform 3 (Q01973-3) contains the extracellular region but with two amino acid substitution at positions 392 and 393 (DS → GK) (69). These ROR receptors were designated orphans because their ligands had not been identified at that time; however, it is now established that Wnt molecules are their main ligands and bind to the Frizzled domain. Thus, signals are transduced using the non-canonical (β-catenin-independent) pathway (9, 72).

Receptor tyrosine kinase-like orphan receptor 1 undergoes several post-translational modifications, such as glycosylation, which generate ROR1 variants with different molecular weights (100, 115, and 130 KDa). Inhibition of ROR1 glycosylation alters the cell surface localization of the 130 KDa variant and the ROR1-induced filopodia formation (73). *ROR1* and *ROR2* are highly express during embryogenesis in most tissues derived from all three germ layers but decrease after birth (67). However, low levels of ROR1 are found in normal adult organs and tissues (74), including the brain, liver, bladder, kidney, ovary, pancreas, bone marrow, adipose tissue, lymphoid and endocrine systems, and gastrointestinal and respiratory tracts (75) (Supplementary Figure 2). After the discovery of RORs, ROR2 became more relevant than ROR1 because mutations in *ROR2* were associated with drastic skeletal alterations in bones formed by endochondral ossification, cardiac abnormalities, and pulmonary dysfunction that resulted in neonatal death (76). In humans, *ROR2* mutations cause brachydactyly type B (77). On the other hand, although human *ROR1* mutations are not associated with morphological alterations, *mRor1*-deficient mice die after birth owing to a respiratory dysfunction (78).

## Relevance of ROR1 as tumor marker

Receptor tyrosine kinase-like orphan receptors play an essential function in some features of tumor cell behavior, such as migration and invasiveness. Recently, in the large quest to discover tumor therapeutic targets, ROR proteins were found to be expressed in human cancers (79). In the last few years, more attention has been paid to ROR1 because its elevated expression in many human neoplasms (breast, ovarian, lung) is associated with cancer development, progression, and metastasis. In fact, a growing number of studies have pointed to ROR1 as a human tumor-associated antigen (TAA) (9, 80, 81). Therefore, ROR1 has been proposed as a potential target for treating these malignancies (9, 10, 70). However, the presence of low levels of ROR1 in normal adult tissues, including parathyroid, pancreatic islets, stomach, and duodenum, should be considered to prevent cytotoxicity (74) (Supplementary Figure 2).

The ROR1 ligand, Wnt5a, is a cytokine highly expressed in cancer tissues. In the triple-negative breast cancer (TNBC) cell line MDA-MB-231, Wnt5a binds to ROR1 to signal and activate the PI3K/AKT/CREB pathway. This pathway controls genes such as *BCL2, CCND1*, and *CCNB1*, which are involved in cancer cell survival and proliferation (9). Hasan et al. (82) evaluated the role of Wnt5a after transfecting *ROR1* in the ROR1-negative (ROR1*^Neg^*) breast cancer cell line MCF7. They observed that in the ROR1^+^MCF7 cells, Wnt5a stimulated the ROR1-dependent cortactin phosphorylation which recruited ARHGEF1 (Rho guanine nucleotide exchange factor 1) to activate RhoA and promote cancer cell migration (82). On the other hand, Zhang et al. (83) showed that chemotherapy can also increase ROR1 levels in breast cancer cells, that, in turn, enhance the expression of genes induced by the activation of RhoGTPases, Hippo-YAP/TAZ, or B lymphoma Mo-MLV insertion region homolog 1 (BMI11). In this study, ROR1 was shown to promote the ability of cancer cells to migrate in Matrigel, engraft in immunodeficient mice, and survive chemotherapy. Moreover, blockade of ROR1 with an anti-ROR1 mAb (cirmtuzumab) reduced the downstream signaling pathway and decreased the invasiveness of cancer cells and their ability to persist after chemotherapy (83). Other anti-ROR1 mAbs, such as zilovertamab, are currently being evaluated to treat ovarian and endometrial cancer (84).

Molecular analyses have confirmed the high expression levels of *ROR1* mRNA in human ovarian cancer biopsies compared to normal ovary samples and suggested ROR1 as a TAA and a promising therapeutic target for this type of neoplasm (81). siRNA silencing of *ROR1* and *ROR2* and their ligand, *WNTA5*, in the ovarian cancer cell line OVCAR3 led to decreased malignant cell proliferation, migration, and invasiveness (85). Patients with ovarian cancer expressing high levels of *ROR1* have a short median survival due to a small population of so-called cancer stem cells (CSCs). These cells are resistant to chemotherapy and exhibit a metastatic phenotype, including high levels of *ROR1*, which makes them suitable targets for anti-ROR1 therapies (7). In a murine model, Wu et al. (86) evaluated a vaccination strategy against epithelial ovarian carcinoma (EOC). For this purpose, they isolated CD117^+^ CD44^+^ CSCs from EOC cell line. These CSCs expressed high levels of *ROR1* and were used to prepare a lysate to be administered subcutaneously to mice. The immune response triggered by the vaccine entailed the production of INF-γ and anti-ROR1 antibodies, inhibited tumor growth, and improved mice survival (86). Furthermore, Zhang et al. (7) working with ROR1^+^ CSCs, isolated from xenografts derived from human primary ovarian tumors, demonstrated that anti-ROR1 mAb UC-961 inhibited the ability of those cells to self-renew, form spheroids or engraft in immunodeficient mice (7). These results point to ROR1 as a relevant therapeutic target for patients with ovarian cancer.

Lung adenocarcinoma cells also express elevated levels of ROR1 when compared to adjacent non-tumor tissue cells. The ROR1 high expression correlates significantly with the attributes of the malignant cells (12), and is associated with a worse overall survival (80). In this cancer, ROR1 downregulates proapoptotic molecules (Bak, Caspase-3, Caspase-7) and upregulates antiapoptotic molecules (Bcl-2 and Bcl-XL) and proteins involved in the cell cycle, such as CDK4 and cyclin E1 (12). *ROR1* silencing in non-small cell lung cancer (NSCLC) cell lines led to decreased activity of the PI3K/AKT/mTOR signaling pathway, which is related to apoptosis and antiproliferative effects on tumor cells (87).

Similarly, human and mouse hepatocellular carcinoma cell lines overexpress *ROR1*. *ROR1* silencing in those cell lines, decreased proliferation and migration but increased resistance to apoptosis (88). Elevated expression of ROR1 and related proteins (pAkt and pCREB) was also observed in biopsies from human gastric adenocarcinoma by immunohistochemical staining. The expression of ROR1 and pCREB was associated with the Ki67 labeling index, pointing to a role for these molecules in tumor cell proliferation (89), similar to observations in human breast cancer cells (9). In neuroblastoma, the highly expressed ROR1 down-regulates the PI3K/AKT-dependent pathway that promotes cell differentiation. In this cancer, retinoic acid, used as a therapeutic agent for over a decade, was shown to bind to the promoters of *ROR1* and *WNTA5* to regulate their transcription, reducing cell proliferation and promoting cell differentiation, as evidenced by the increased expression of synaptophysin (90). Several studies have provided evidence for the relationship between the PI3K/AKT signaling pathway and the role of ROR1 on tumor cell progression and invasiveness (87, 91–93). Therefore, proteins downstream of that signaling pathway could be relevant therapeutic objectives for future cancer treatments.

Epithelial-mesenchymal transition (EMT) is a common feature of metastatic cells which are characterized by overexpressing ROR1. In the metastatic breast cancer cell line MDA-MB-231, suppression of ROR1 function led to reduced expression of vimentin, SNAIL-1/2, and ZEB1, but increased levels of E-cadherin, CK-19, and ZO-1; in addition, the ability of MDA-MB-231 cells to migrate and invade decreased. However, *ROR1* transfection in ROR1*^Neg^* MCF7 cells showed some opposite effects, specifically, increased expression of vimentin and SNAIL-1/2. Treatment with the anti-ROR1 mAb D10, could modulate and inhibit the migration of these cancer cells *in vitro* and *in vivo* (94). Similarly, ROR1 is overexpressed in melanoma cells, enhancing AKT activation and participating in the EMT process by increasing the expression of the mesenchymal markers, vimentin, and N-cadherin (91). Interestingly, circulating tumor cells (CTCs) from patients with pancreatic cancer (PAC) showed higher levels of ROR1 and greater proliferative and invasive potential compared to cells from non-cancerous tissues and the PAC cell lines, PANC-1 and SW-1990. CTCs, considered functional markers of metastasis, were characterized by the up-regulation of N-cadherin and the downregulation of E-cadherin. These data support a role for ROR1 in the EMT, a process extensively reviewed by Loh et al. (95, 96). *ROR1* knockdown restored E-cadherin levels, increased N-cadherin expression, and inhibited migration and invasiveness of PAC CTCs (96). *ROR1* expression is not limited to solid tumors. It is also present in hematopoietic cancers such as B-ALL (97), chronic lymphocytic leukemia (CLL) (98), mantle cell lymphoma (MCL) (99), and diffuse large B-cell lymphoma (DLBCL) (93). In CLL, *ROR1* is expressed in isoforms of different molecular weights (64, 105, 130, and 260 kDa), constitutively phosphorylated at both tyrosine and serine residues. This phosphorylation is more prominent in progressive than non-progressive disease. The 64 kDa ROR1 isoform localizes within the nucleus, probably regulating gene transcription. Blocking ROR1 phosphorylation with mAbs drove cancer cells into apoptosis (98). The 260 kDa ROR1 isoform results from the homodimerization of ROR1 or the heterodimerization with ROR2 (98). The latter increases cell proliferation and migration of CLL cells by a mechanism mediated by the binding of Wnt5a (100).

## ROR1 CAR-T cells under investigation

A therapeutic alternative for patients with cancer that has become relevant in the last years is the treatment with *ex vivo* genetically engineered autologous lymphocytes. Adoptive immunotherapy based on CAR-T cell infusion is a promising approach to target tumor antigens expressed on the tumor cell membrane (101). Optimizing CAR-T cell therapy requires knowing the antigenic profile expressed by tumor cells and their analysis in the context of normal tissues. This knowledge will improve therapy results and avoid injury to self-tissues (102).

The typical architecture of a second-generation CAR comprises scFv, hinge, transmembrane, and intracellular regions. The scFv region is the domain for tumor antigen recognition. It usually derives from the variable domains of a monoclonal antibody’s heavy and light chains which are coupled to a short linker. The hinge region is a connecting peptide that provides the CAR with the conformational freedom necessary to facilitate its binding to the target antigen on the tumor cell; it can be short or long. The transmembrane (Tm) region come from CD8, CD28, or another co-stimulatory domain. The intracellular region comprises co-stimulatory domains (CD28 or 4-1BB) and a CD3ζ domain that triggers intracellular signaling for CAR-T cell activation, expansion, cytokine synthesis, and cytotoxic activity (101).

**FIGURE 2:**
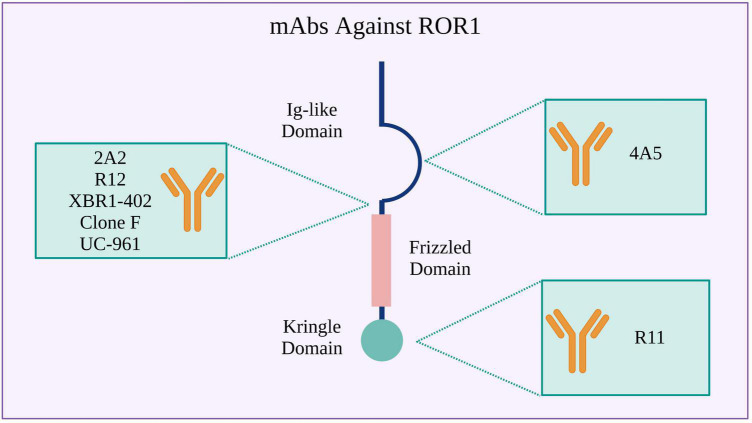
Monoclonal antibodies (mAbs) used to design ROR1 CARs. Binding sites in the ROR1 extracellular region are shown.

**FIGURE 3:**
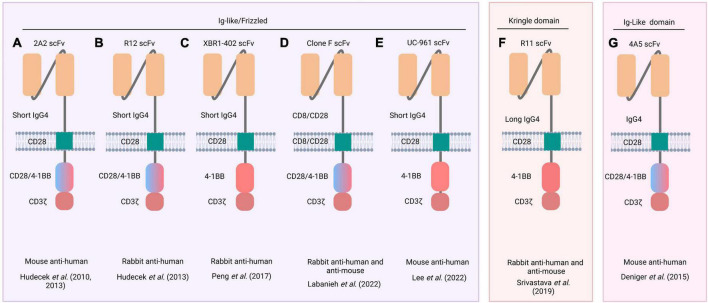
Schematic representation of ROR1 CARs. Five different scFvs (derived from 2A2, R12, XBR1-402, clone F, and UC-96 mAbs) recognize an epitope between the Ig-like and Frizzled domains of ROR1 (left inset) **(A–E)**, whereas R11 recognizes an epitope in the Kringle domain (middle inset) **(F)**, and 4A5 only recognizes an epitope in the Ig-like (right inset) **(G)**. Names of the source mAbs appear at the top of each CAR structure. The blue/pink double-colored intracellular region indicates that two CARs were constructed, one with the CD28 costimulatory domain and another with the 4-1BB. The animal species from which mAb were obtained and the respective reference are listed below each CAR structure.

Receptor tyrosine kinase-like orphan receptor 1 CARs can be assembled using scFvs derived from various mAbs developed for treating hematological and solid tumors. The 2A2 (103), R12 (53), XBR1-402 (99, 104), clone F (105), and UC-961 (106) mAbs bind to an epitope at the N-terminal ROR1 region between the Ig-like and Frizzled domains. In turn, the 4A5 (107) and R11 (108) mAbs bind to specific epitopes in the Ig-like and Kringle domains, respectively (Figures 2, 3 and Supplementary Table 1). The CARs manufacturing can be achieved through different ways for CAR gene delivery, for example using lentiviral non-replicating vectors (LVs) or transposable elements.

### ROR1 CAR gene delivery using non-replicating LVs

Hudecek et al. (103) engineered a CD8^+^ CAR-T cell using the scFv derived from the anti-ROR1 2A2 mAb. The CAR structure included an IgG4 hinge domain, a CD28 Tm domain, and a CD28 or 4-1BB co-stimulatory domain followed by a CD3ζ domain (103). These CAR-T cells were cocultured at different ratios with tumor cells from B-cell CLL or MCL to measure their cytotoxic effect. At all cell ratios tested, ROR1 CAR-T cells destroyed ROR1^+^ tumor cells but not normal cells, showing the specificity of this type of therapy (103) (Figure 3A).

Subsequently, Hudecek et al. (103) demonstrated the relevance of the hinge length in enhancing the cytotoxic effect of CAR-T cells. They designed ROR1 CARs containing scFvs derived from 2A2 and R12 mAbs coupled to a hinge domain (IgG4-Fc spacer) of different lengths (long: 229 aa, intermediate: 119 aa, or short: 12 aa), followed by a human Tm CD28 domain, and a cytoplasmic region (human CD28 or 4-1BB bound to CD3ζ) (53) (Figures 3A, B). They then evaluated the effector activity of these ROR1 CAR-T cells against ROR1^+^ tumor cells. 2A2 ROR1- and R12 ROR1-CAR-T cells engineered with a short-length hinge domain showed more significant cytotoxic effects and cytokine production (IFN-γ, TNF-α, and IL-2) than CAR-T cells with long-length hinge domains. These results suggested that the shorter the hinge domain, the better the spatial coupling of CAR-T cells to the ROR1^+^ target cells and the subsequent activation of T-cells (53). Furthermore, R12 ROR1-CAR-T cells containing a short hinge domain showed greater effector functions than 2A2 ROR1-CAR-T cells with a short hinge domain (109, 110). Therefore, the length of the hinge domain and the receptor affinity should be considered in CAR design. Wallstabe et al. (50) observed similar results for 2A2 ROR1- and R12 ROR1-CAR-T cells with short hinge domains when evaluated against A549 (lung) and MDA-MB-231 (breast) cancer cell lines cultured in a 3D model (50). Moreover, Berger et al. (51) observed no significant cytotoxic effect on normal tissues when R12 ROR1-CAR-T cells harboring cytoplasmic 4-1BB and CD3ζ domains were infused intravenously into *Macaca mulatta* (51).

The XBR1-402 CAR (Figure 3C) was constructed with a short IgG4-derived spacer, the Tm domain of CD28, and the signaling 4-1BB, and CD3ζ domains. When T-cells engineered to express this CAR were co-cultured with the ROR1^+^ breast cancer cell line MDA-MB-231, they exhibited rapid proliferation, high levels of IFN-γ and IL-2, and the ability to kill the tumor cells (104). Notably, XBR1-402 ROR1-CAR-T cells were found to be equally or more potent than R12 ROR1-CAR-T cells with the same short spacer and signaling domains (104).

Following CAR-T cell infusion, some life-threatening side effects may occur. The most common are fever, anaphylaxis, weight loss, neuronal toxicity, respiratory distress, and cytokine release syndrome (CRS) (108, 111, 112). To avoid this toxicity resulting from shared antigen expression between tumor and normal cells, new methods such as sensitive tuning of CARs to antigen density (113), CARs regulated by inhibitory molecules (105), and combinatorial antigen detection circuits (108, 114) are employed. A drug-regulated expression system named Signal Neutralization by an Inhibitory Protease (SNIP) has been developed to regulate CAR-T cell activity. For example, ROR1(F).28z CAR-T cells (Figure 3D) (105) exhibit lung and liver toxicity in a murine model. Therefore, SNIP ROR1(F).28z CAR-T cells were engineered. This construct comprised the scFv fragment derived from clone F mAb (115) followed by the hinge, Tm, co-stimulatory (CD28), and signaling (CD3ζ) domains and a cleavage site for the hepatitis C virus NS3 protease (NS3p) between the Tm and signaling domains. In addition, these T-cells were engineered to express a membrane-bound NS3p with a Tm (CD28) domain. Thus, T-cells expressing these constructs were apt to be regulated by adding a specific protease inhibitor. When no inhibitor is added (OFF state), the CAR activity is drastically reduced because the NS3p cleaves the CAR structure. In contrast, in the presence of the inhibitor (ON state) the NS3p is inactive, and CAR activity is triggered. In mice bearing live ROR1^+^ Nalm6 leukemic cells, conventional ROR1(F).28z CAR-T cells were more active but elicited a lethal effect. However, SNIP ROR1(F).28z CAR-T cells tuned with appropriate doses of the NS3p inhibitor caused no toxic effect while maintaining antitumor activity, resulting in high safety and efficient therapeutic outcomes.

Lee et al. (106) designed CAR-T cells using the scFv derived from zilovertamab (UC-961), followed by a short hinge segment (IgG4), a Tm (CD28), and the intracellular 4-1BB and CD3ζ domains (Figure 3E) (106). These CAR-T cells showed *in vitro* antitumor activity against lung and breast cancer cell lines. *In vivo*, they significantly suppressed tumor xenograft growth and produced no important side effects (106).

Srivastava et al. (108) constructed the R11 ROR1 CAR, which recognizes an epitope in the Kringle domain of ROR1 conserved in mice and humans. This R11 ROR1 CAR construct was assembled with a long hinge segment (modified human IgG4), a Tm (murine CD28) domain, and intracellular 4-1BB and CD3ζ domains (108) (Figure 3F). Authors showed that R11 ROR1-CAR-T cells caused mouse death due to the recognition of ROR1^+^ normal cells in bone marrow and spleen. Accordingly, they then applied the synthetic Notch receptor (synNotch) strategy to overcome the adverse cytotoxic effect. For this purpose, R11 ROR1-CAR-T cells were further transfected to express a synNotch receptor directed against a different tumor antigen (epithelial cell adhesion molecule (EpCAM) or B7-H3) expressed exclusively on ROR1^+^ tumor cells. Recognition of EpCAM or B7-H3 on ROR1^+^ tumor cells activated the synthesis of a transcription factor that, in turn, promoted the transcription of the ROR1 CAR gene. Thus, the cytotoxic activity of CAR-T cells was directed only against tumor cells in which both EpCAM (or B7-H3) and ROR1 antigens were present, with no detrimental effect on normal ROR1^+^ cells (108) (Supplementary Table 1).

### ROR1 CAR gene delivery using transposable elements

In cell therapy, CAR gene delivery using LVs faces problems related to the formation of replication-competent viral vectors, mutational integration of the provirus into the host cellular genome, and mobilization of structural viral genes to target cells. Moreover, developed LVs derived from the human immunodeficiency virus (HIV) poses further safety concerns (116). Thus, to overcome this problem, some research is looking for different ways to the intracellular release of CAR genes. The *Sleeping Beauty* (SB) transposon system is an alternative platform for CAR-T cell manufacturing (117, 118), which seems to address the need for a non-viral gene transfer approach that could reduce the cost and avoid the manufacturing issues associated with the transduction of T-cells with recombinant viral vectors and accelerate the translation of preclinical data into clinical trials (117, 119–121).

The SB system was used by Deniger et al. (107) to insert the heavy and light chain sequences of the 4A5 mAb into a ROR1 CAR construct containing the CD28/CD3ζ or 4-1BB (CD137)/CD3ζ costimulatory/signaling domains. They observed that T-cells expressing the CAR coupled to 4-1BB/CD3ζ showed more effective antitumor activity (107) (Figure 3G). Huang et al. (122) also used the SB system to engineer T-cells to express a ROR1 CAR comprising the scFv from the 2A2 mAb, a Tm domain (CD28), and the 4-1BB and CD3ζ domains. These ROR1 CAR-T cells were effective against sarcoma cells as evidenced by the synthesis of high levels of IFN-γ, TNF-α, and IL-13 in cocultures with sarcoma cell lines and the ability to reduce the tumor growth in a murine osteosarcoma xenograft model (122) (Supplementary Table 1).

## Methods employed to evaluate ROR1 CAR-T cell function

*In vitro* methods are the most widely used in research because they make it possible to elucidate cellular and molecular mechanisms in a controlled manner. Two-dimensional (2D) cell cultures support cell growth in monolayers but do not recreate the conditions observed *in vivo*. In turn, 3D cell cultures more accurately recreate *in vivo* cellular processes such as gene expression, cell organization, differentiation, and signaling, as well as cell-cell and cell-extracellular matrix interactions (123, 124).

Chimeric antigen receptor cell therapy has been successful in treating hematological malignancies (125, 126) but remains challenging for solid tumors. Advancing research to improve CAR-T cell performance is critical in therapies designed to treat this kind of tumor, where factors such as tumor infiltration by T-cells, immunosuppressive TME, and tumor cell heterogeneity hinder the desired clinical outcome. Therefore, further investigation of these factors is necessary to promote the clinical approval of CAR-T cells against solid tumors (16). For this reason, it is essential to use *in vitro* models that can mirror existing *in vivo* conditions to understand CAR-T cell function and the role of TME in therapy outcomes (17, 18, 50). Various 3D cell-culture systems, such as organoids, spheroids, bioprinting, and microdevices have been used to assess CAR-T cell function (50, 127–129).

Yuki et al. (17) and Boucherit et al. (18) reviewed 3D cell-culture systems developed on Matrigel or collagen platforms using chopped tumor samples or tumor cells in suspension to evaluate anti-cancer therapies such as immune checkpoint blockade, TILs generation, biomarker-based patient response prediction, and exploration of tumor therapeutic targets to improve clinical outcome. These studies have corroborated the key role played by TME in the antitumor immune response by altering the phenotype and function of immune cells (17, 18). Thus, 3D cell-culture systems could be employed to assess CAR-T cell functionality.

Hudecek et al. (103) demonstrated that the cytotoxic activity of ROR1 CAR-T cells could be evidenced when cocultured with suspensions of ROR1^+^ tumor cells (Jeko-1, Rec-1, BALL-1, RCH-ACV, and RPMI-8226) (103). Similar cytotoxic effects were observed in 2D cocultures of ROR1 CAR-T cells with adherent ROR1^+^MDA-MB-231 cancer cells (104).

Wallstabe et al. (50) evaluated ROR1 CAR-T cell function using a 3D cell-culture system made of decellularized porcine jejunum functioning as a scaffold with an intact basement membrane. A549 or MDA-MB-231 cancer cells were seeded on the luminal side of the scaffold and generated a progressively increasing cell mass with an invasive phenotype. When ROR1 CAR-T cells were added, they could infiltrate and migrate into this 3D tumor system exerting a cytotoxic effect that killed cancer cells. However, they showed phenotypic markers of cellular exhaustion (50). In the same model, Stuber et al. (24) showed that the addition of TGFβ significantly decreased the cytotoxic/antitumor activity of ROR1 CAR-T cells on MDA-MB-231 cancer cells. Furthermore, treatment with SD-208, a TGFβ inhibitor, prior to TGFβ exposure restored the effector activity of CAR-T cells to levels similar to those observed in cultures non-exposed to TGFβ (24).

Murine models (wild-type, immunodeficient, gene-specific knockdown strains) have also been used to evaluate CAR-T cell therapy. In many of these studies, mice are inoculated with bioluminescent cancer cells, and tumor size is tracked over time (53, 105, 122). For example, in the study conducted by Srivastava et al. (108) to evaluate the SynNocht strategy (previously described) to avoid toxicity and death caused by ROR1 CAR-T cells, different mouse strains such as C57BL/6J (B6), BALB/c Rag2^–/–^, ROR1-KO were used in the different experiments (108). Subsequently, Srivastava et al. (130) used a genetically engineered mouse model of human lung adenocarcinoma adapted to express ROR1. They showed that combined treatment with cyclophosphamide, oxaliplatin, CAR-T cells, and anti-PD-L1 improved CAR-T cell-mediated tumor control. The model allowed them to evidence that oxaliplatin activated tumor-infiltrating macrophages to secrete T-cell recruiting chemokines, thus resulting in increased CAR-T cell infiltration, remodeling of the TME, and increased tumor sensitivity to anti-PD-L1 (130).

Tumor xenografts are another strategy used to evaluate CAR-T cells *in vivo.* In this approach, cell lines derived from primary human tumors are transplanted into immunodeficient mice. For example, in the SNIP ROR1 CAR-T cell study (described above), Labanieh et al. (105) tested CAR-T cell activity in NGS (NOD SCID *Ilgr2* null) mice transplanted with Nalm6 ROR1^+^ leukemic cells. At 1-day post-tumor engraftment, SNIP ROR1 CAR-T cells were injected intravenously, and NS3p inhibitor was administered only for 2 days. This protocol showed a reduction in tumor size without animal lethality (105).

*Macaca mulatta*, a non-human primate, is a promising model for evaluating the effects of ROR1 CAR-T cells because ROR1 is highly conserved between humans and macaques and has a similar tissue expression pattern. A study showed that autologous infusion of ROR1 CAR-T cells did not cause significant adverse effects. Specifically, there were no changes in body weight, complete blood cell counts, serum chemistry (pancreatic, liver, and muscle enzymes) or plasma levels of IFN-γ, IL-6, and IL-12 (51).

## ROR1 CAR-T cells employed in clinical trials

Approximately 1,314 clinical trials of CAR-T cell therapies (https://clinicaltrials.gov/) are ongoing for managing leukemias, lymphomas, and solid tumors. These CAR-T cells target different antigens, but ROR1 was the tumor antigen selected in just five studies, namely, two for hematological malignancies (NCT02194374 and NCT05588440), two for solid tumors (NCT05274451 and NCT05638828), and one for both (NCT02706392) (131).

The NCT02706392 trial conducted by the Fred Hutchinson Cancer Center was a Phase I trial intended to treat patients with ROR1^+^ cancers. Malignancies included in the study were CLL, MCL, ALL, stage IV NSCLC, and TNBC. This trial was focused on finding the optimal dose of CAR-T cells and determining the side effects of the therapy. The study started in 2016 and has now been completed (132). Preliminary results showed that patients with TNBC did not present severe neurotoxicity or severe CRS after infusion of increasing doses (3.3 × 10^5^, 1 × 10^6^, 3.3 × 10^6^, and 1 × 10^7^ cells/kg) of ROR1 CAR-T cells. Moreover, the disease remained stable after CAR-T cell infusion in two patients at 15 and 19 weeks, respectively. Following the first CAR-T cell infusion, one patient showed stable disease, and after the second infusion, a confirmed partial response was observed and persisted for 14 weeks (133). A report on this study by Srivastava et al. (130) using ROR1 CAR-T cells manufactured carrying R12 scFv, 4-1BB and CD3ζ signaling domains showed that in three patients (ROR1^+^ TNBC or ROR1^+^ NSCLC), the ROR1 CAR-T cells expanded as evidenced in peripheral blood. However, the engineered cells poorly infiltrated the tumors and displayed reduced functionality; moreover, no tumor regression was observed (130). The poor tumor infiltration and low persistence of ROR1 CAR-T cells may be related to the immunosuppressive TME that leads the engineered T-cells to upregulate multiple inhibitory receptors and loss the capacity to produce interferon-γ (IFN-γ), tumor necrosis factor-α (TNF-α), and granulocyte-macrophage colony-stimulating factor (GM-CSF) (130).

The NCT05274451 trial, a Phase I study, was designed by Lyell Immunopharma, Inc. Its objective is to evaluate the safety and tolerability of LYL797, a ROR1 CAR-T cell product in patients with relapsed or refractory TNBC and NSCLC. It started in 2022, is currently in the patient recruitment phase, and is estimated to be completed in 2026 (134). The LYL797 CAR expresses the scFv derived from the R12 mAb and was constructed with Gen-R and Epi-R technologies. Gen-R modifies cells to overexpress *c-Jun*, resulting in less cellular exhaustion, and Epi-R maintains a stem cell-like phenotype that enhances the functional activity of CAR-T cells (135).

The third trial, NCT02194374, was designed by the M.D. Anderson Cancer Center. It focused on the treatment of patients with CLL, started in 2015, but was closed in 2017 with no patient enrollment or results (136). The NCT05638828 trial at Peking University started in 2022 and is in the patient recruitment phase. This early phase 1 study aims to evaluate the safety and tolerability of escalating doses of the ROR1 CAR-T cells called RD14-01 in patients with advanced ROR1^+^ solid tumors (137). Finally, the NCT05588440 trial is a phase 1/2 study conducted by Oncternal Therapeutics, Inc. It aims to investigate the safety and efficacy of the CAR-T therapy, ONCT-808, in patients with relapsed/refractory (R/R) aggressive B-cell malignancies and is currently enrolling patients (138).

## Promising combined therapeutic approaches

Combined therapeutic strategies are used to treat patients with cancer in order to achieve the best outcomes. The most conventional consist of surgery followed by radiotherapy and/or chemotherapy. Currently, other therapeutic approaches are designed to act on a specific alteration (gene, receptor) of neoplastic cells; one of them is the immunotherapy directed against a single TAA. However, this strategy has the disadvantage of exerting a selective antigenic pressure that results in the proliferation of malignant cells with low or no expression of the target antigen, which translates into their resistance to conventional treatments (7). For instance, CD19 CAR-T cells (Kymriah^®^, Yescarta^®^, Tecartus^®^, Breyanzi^®^, and ARI-0001) (139) have been successful in treating patients with B-cell malignancies (140); however, relapses related to down-regulation of the target antigen occurred in 25% of cases (141, 142). In addition, tumor antigens can escape immune strategies by other mechanisms, such as receptor genetic mutation, epitope masking, and trogocytosis (142–144). Therefore, simultaneous targeting of multiple tumor antigens or combining immunotherapy with other anti-cancer drugs could help prevent antigenic escape and reduce patient relapse. Gill et al. (145) observed synergy between CD19 CAR-T cells and ibrutinib in treating patients with CLL; 72% of individuals had no detectable residual disease after 12 months (145). On the other hand, multiple antigen targeting CAR constructs comprise a dual CAR construct or a tandem construct (two single-chain variable fragments) (146, 147). CD19/CD22 and CD19/BCMA CAR-T cells are current therapeutic strategies for targeting multiple antigens that have shown promising results (148, 149). Shah et al. (150) designed bispecific CD20-CD19 CAR-T cells to treat patients with B-cell malignancies and observed a rapid response at 28 days post-infusion; 64% of patients had a complete response whereas 18% had a partial response (150).

The heterogeneity of solid tumors has hindered the identification of robust and suitable targets for CAR-T cell therapy (139). Moreover, the occurrence of some antigenic targets in normal tissues further complicates the task (74, 75). Yang et al. (151) produced bivalent tandem CAR-T cells directed against CD70 and B7-H3, two antigens co-expressed in some lung and breast cancers and melanoma. Compared to conventional CAR-T cells, tandem CAR-T cells showed a higher cytotoxic effect and increased cytokine production in co-cultures with cancer cell lines and after being infused into lung and melanoma xenograft-bearing animals (151). In turn, Srivastava et al. (108) used a logic-gated CAR-T cell therapy in which ROR1 CAR was combined with a SynNocht receptor against a different tumor antigen (EpCAM or B7-H3) to treat animal models of human breast cancer. As previously described, these SynNocht ROR1 CAR-T cells avoided the adverse cytotoxic effects observed with conventional ROR 1 CAR-T cells (108).

Lung and breast cancers are malignant neoplasms that affect many people worldwide (1). Some surface antigens expressed in both malignancies are promising therapeutic targets. In lung cancer, the most relevant therapeutic targets include ROR1, EGFR, PD-L1, CD80/CD86, mucin 1 (MUC1), mesothelin (MSLN), carcinoembryonic antigen (CEA), prostate stem cell antigen (PSCA) and human epidermal growth factor receptor 2 (HER2) (139, 152). For breast cancer, the most common therapeutic targets are CEA, CD70, ROR1, HER2, MUC1, EGFR, CD133, MSLN, EpCAM, disialoganglioside (GD2), and the hepatocyte growth factor receptor (HGFR) (153, 154).

EGFR CAR-T cells have been evaluated *in vitro* and *in vivo*. In cocultures with EGFR^+^ NSCLC cell lines, EGFR CAR-T cells exerted a cytotoxic effect after 3 h-incubation and produced elevated levels of IL-2, IL-4, IL-10, tumor necrosis factor (TNF-α), and IFN-γ (155). *In vivo*, EGFR CAR-T cells also showed activity after infusion into animals bearing human lung cancer xenografts; specifically, they induced tumor regression at 25 days, as evidenced by bioluminescence imaging that showed a reduction in tumor size (155). EGFR CAR-T cells have been used in patients with refractory NSCLC at escalating doses. In this case, tumor biopsies showed the elimination of EGFR^+^ tumor cells and EGFR CAR-T cell infiltration. Furthermore, no severe side effects were observed (156). EGFR CAR-T cells also have shown activity against TNBC. *In vitro* and *in vivo*, they exhibit cytotoxic effects, produce high levels of cytokines, and inhibit tumor growth. Moreover, EGFR CAR-T cells persist, infiltrate tumors, and activate apoptosis pathways with minimal side effects (157–159).

Considering that EGFR is overexpressed in 40–89% of NSCLC (160) and 5–30% of breast cancer tumors (161, 162), the described scenario places EGFR as a candidate for therapeutic strategies that combine EGFR and ROR1 CAR-T cells. CAR-T cell therapy against two different antigens could improve the clinical outcome in patients with these solid tumors.

In conclusion, the CAR-T cell approach for treating patients with solid tumors remains challenging, given tumor heterogeneity and inhibitory signals derived from the TME. However, some advances in the treatment of solid tumors are currently employing the ROR1 antigen as a critical target due to its high expression in tumor cells and relatively scarcity in normal tissues, as well as its presence in CSCs responsible for relapses and treatment resistance. In addition, anti-ROR1 mAbs are available, and new methods to produce ROR1 CAR-T cells promise to be less harmful to patients. In addition, some strategies developed for other tumor therapeutic targets could help improve the functionality of different ROR1 CAR-T cells and, consequently, the clinical outcomes of this cancer immunotherapy. These approaches include combining scFv of different affinities, using hinge segments of different lengths, and incorporating various costimulatory domains into the CAR gene to increase the tuning and potency of CAR-T cells. Moreover, given the broad expression of some TAAs in different normal tissues, new CAR designs should include strategies to guarantee treatment safety. In this regard, a better selection of tumor-specific antigens will allow focus treatment on tumor cells. Another alternative to overcome the problems associated with widely expressed TAAs is to combine two low- or moderate-affinity scFvs in a single CAR-T cell to achieve a more specific target tuning. Finally, combining CAR-T cells with other therapies, such as blocking TME inhibitory signals, could improve clinical outcomes.

## Author contributions

DO-R and CR-S drafted and edited the manuscript. All authors reviewed, contributed to intellectually, and approved the submitted version.

## Supplementary material

Supplementary Figure 1 Sequence comparison of human vs. mouse ROR1 **(A)** and alignment of different human ROR1 isoform sequences **(B)**. Signal peptide (M1-A29) – extracellular (Q20-Y406) – transmembrane (I407-C427) and – cytoplasmic domains (V428-L/V937).

Supplementary Figure 2 *ROR1 RNA* expression in normal tissues. The summary shows the consensus data bases on normalized expression (nTPM) values from two different sources, the human protein atlas RNA-seq data and RNA-seq data from the genotype-tissue expression project (https://www.proteinatlas.org/ENSG00000185483-ROR1/tissue) (62).
