# Supplementary material for: Anti-ROR1 CAR-T cells: Architecture and performance
Source: Front Med (Lausanne). 2023 Feb 17;10:1121020. doi: 10.3389/fmed.2023.1121020 (PMC9981679; doi:10.3389/fmed.2023.1121020)
Supplement: Supplementary file 1 [file Table_1.pdf]

Supplementary Table 1. List of CAR-T cells developed against ROR1.

Gene Delivery Method	scFv	mAb Host	Recognition on ROR1	Co-stimulatory Domains	Reference
LVs	2A2	Mouse	Ig-Like/Frizzled	CD28 or 4-1BB	(53,103)
	R12	Rabbit	Ig-Like/Frizzled	CD28 or 4-1BB	(53)
	XBR1-402	Rabbit	Ig-Like/Frizzled	4-1BB	(104)
	Clone F	Rabbit	Ig-Like/Frizzled	CD28 or 4-1BB	(105)
	UC-961	Mouse	Ig-Like/Frizzled	4-1BB	(106)
	R11	Rabbit	Frizzled	4-1BB	(108)
SB	4A5	Mouse	Ig-like	CD28 or 4-1BB	(107)
	2A2	Mouse	Ig-Like/Frizzled	4-1BB	(122)
