# Supplementary figures and images for: Anti-ROR1 CAR-T cells: Architecture and performance
Source: Front Med (Lausanne). 2023 Feb 17;10:1121020. doi: 10.3389/fmed.2023.1121020 (PMC9981679; doi:10.3389/fmed.2023.1121020)

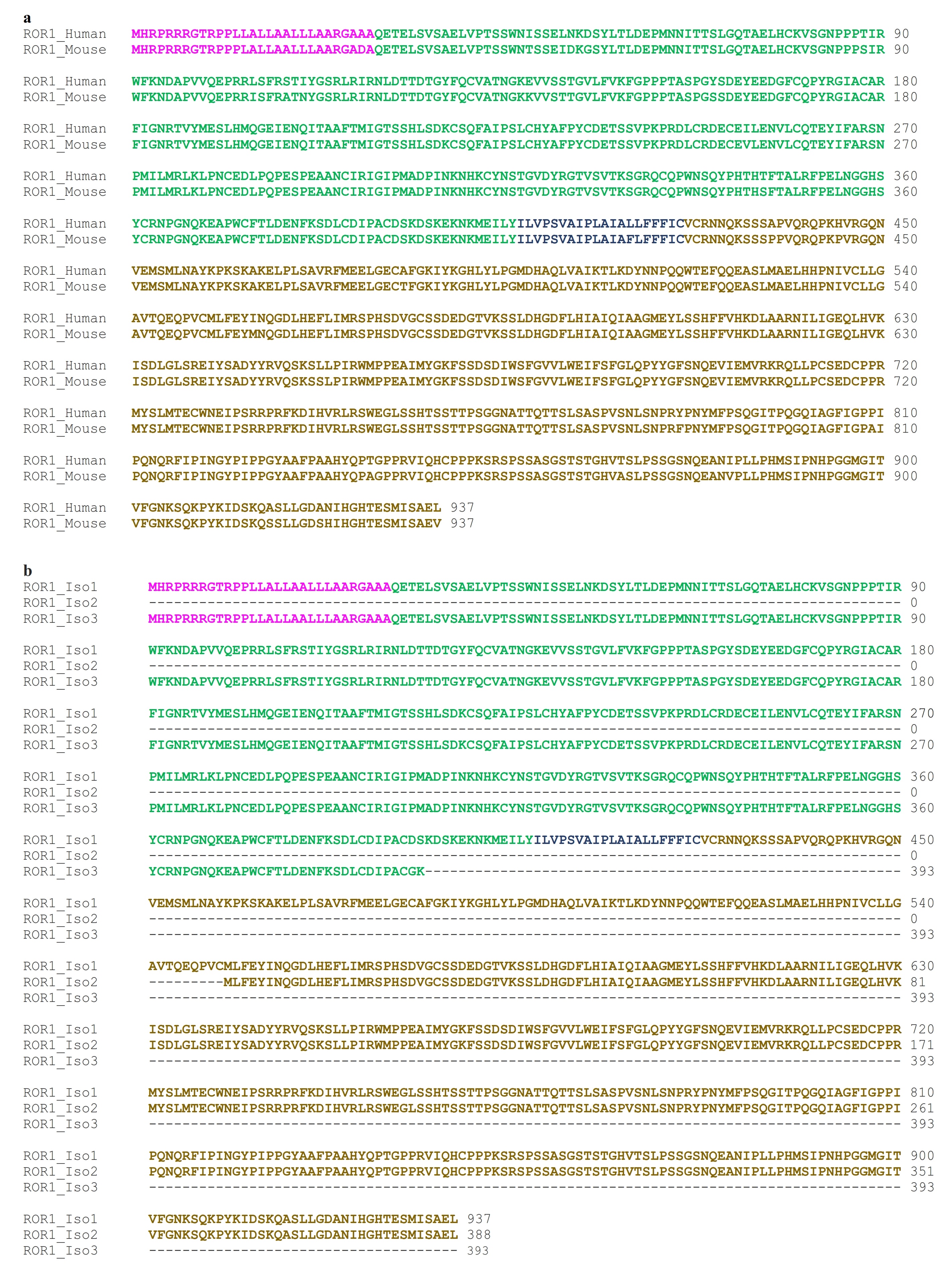

Supplement: Supplementary Figure 1 — Sequence comparison of human vs. mouse ROR1 (A) and alignment of different human ROR1 isoform sequences (B). Signal peptide (M1-A29) – extracellular (Q20-Y406) – transmembrane (I407-C427) and – cytoplasmic domains (V428-L/V937). [file Image_1.jpeg]

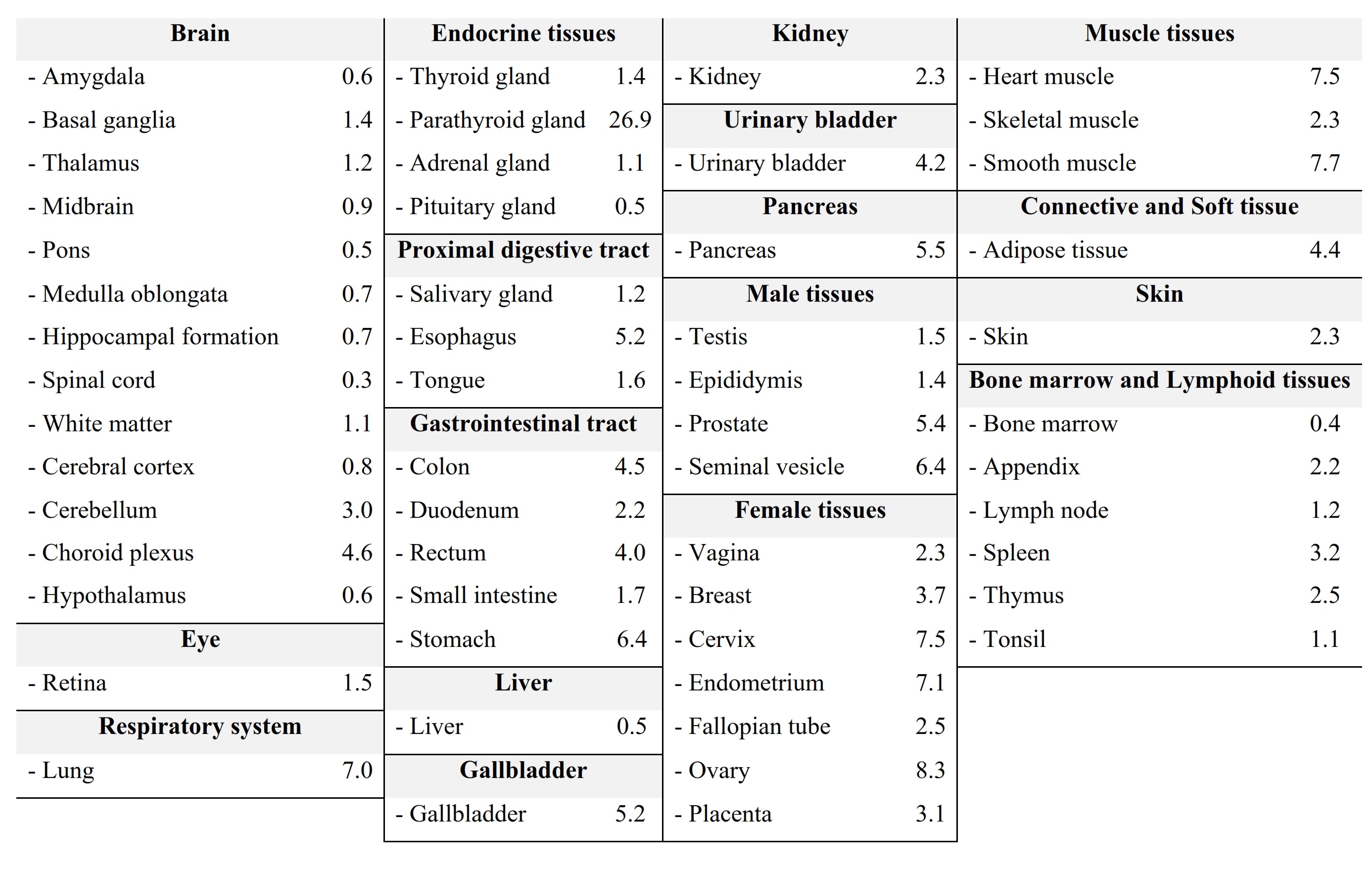

Supplement: Supplementary Figure 2 — ROR1 RNA expression in normal tissues. The summary shows the consensus data bases on normalized expression (nTPM) values from two different sources, the human protein atlas RNA-seq data and RNA-seq data from the genotype-tissue expression project (https://www.proteinatlas.org/ENSG00000185483-ROR1/tissue) (62). [file Image_2.jpeg]
